# Supplementary material for: MetaQTL: a package of new computational methods for the meta-analysis of QTL mapping experiments
Source: BMC Bioinformatics. 2007 Feb 8;8:49. doi: 10.1186/1471-2105-8-49 (PMC1808479; doi:10.1186/1471-2105-8-49)
Supplement: Additional File 7 — MetaQTL Package : jar file and tutorial. This Zip archive contains both the MetaQTL JAR file and the files of the tutorial. [file 1471-2105-8-49-S7.zip › org.inra.metaqtl/doc/org/thalia/bio/class-use/IBioIndividual.html]

Uses of Interface org.thalia.bio.IBioIndividual


|  |  |  |
| --- | --- | --- |
| **Overview** \| **Package** \| **Class** \| **Use** \| **Tree** \| **Deprecated** \| **Index** \| **Help** | |  |
| PREV   NEXT | **FRAMES**    **NO FRAMES**     **All Classes** |  |


---


## **Uses of Interface org.thalia.bio.IBioIndividual**

| Packages that use IBioIndividual | |
| --- | --- |
| **org.thalia.bio** |  |
| **org.thalia.bio.entity** |  |

Uses of IBioIndividual in org.thalia.bio

| Methods in org.thalia.bio that return IBioIndividual | |
| --- | --- |
| `IBioIndividual` | `IBioPopulation.getIndividual(java.lang.String name)` |
| `IBioIndividual[]` | `IBioCross.getParents()` |
| `IBioIndividual[]` | `IBioIndividual.getParents()`             Returns the pedigree of the individual |
| `IBioIndividual[]` | `IBioPopulation.individuals()` |

| Methods in org.thalia.bio with parameters of type IBioIndividual | |
| --- | --- |
| `void` | `IBioPopulation.addIndividual(IBioIndividual individual)` |

Uses of IBioIndividual in org.thalia.bio.entity

| Fields in org.thalia.bio.entity declared as IBioIndividual | |
| --- | --- |
| `IBioIndividual[]` | `Population.parents` |

| Methods in org.thalia.bio.entity that return IBioIndividual | |
| --- | --- |
| `IBioIndividual` | `IndividualContainer.getIndividual(java.lang.String name)` |
| `IBioIndividual[]` | `CrossPopulation.getParents()` |
| `IBioIndividual[]` | `Individual.getParents()` |
| `IBioIndividual[]` | `Population.getParents()` |
| `IBioIndividual[]` | `IndividualContainer.individuals()` |

| Methods in org.thalia.bio.entity with parameters of type IBioIndividual | |
| --- | --- |
| `void` | `IndividualContainer.addIndividual(IBioIndividual individual)` |

---


|  |  |  |
| --- | --- | --- |
| **Overview** \| **Package** \| **Class** \| **Use** \| **Tree** \| **Deprecated** \| **Index** \| **Help** | |  |
| PREV   NEXT | **FRAMES**    **NO FRAMES**     **All Classes** |  |


---
